# Flexible Piezoresistive Sensor Based on Porous PDMS/Candle Soot Foam

**DOI:** 10.3390/s26041086

**Published:** 2026-02-07

**Authors:** Jiaqi Sun, Yanyan Dong, Qi Li, Chenxia Li

**Affiliations:** School of Optical and Electronic Technology, China Jiliang University, Hangzhou 310018, China

**Keywords:** flexible pressure sensor, candle soot, porous PDMS

## Abstract

Flexible wearable pressure sensors still face the challenges of complex structure and high manufacturing costs. In this article, we present a simple method for preparing a highly sensitive, flexible wearable pressure sensor based on candle soot and porous PDMS foam. Meanwhile, to enhance the sensor’s robustness and practicality, a fully enclosed packaging design based on PDMS film was developed. The resulting sensor demonstrates excellent sensitivity, attributed to its porous structure, rough surface, and the unique properties of candle soot. Furthermore, the developed sensor can accurately detect movements in various parts of the human body and measure the force applied during finger pressing. This innovative porous PDMS/candle soot pressure sensor shows great potential for applications in wearable electronics.

## 1. Introduction

In recent years, flexible wearable pressure sensors have garnered significant attention from researchers due to their various applications [1,2,3,4,5]. The prepared flexible pressure sensors can be divided into many types, such as traditional capacitive [6,7,8,9,10] and piezoresistive [11,12,13,14,15] pressure sensors, as well as emerging self-powered [16,17,18,19,20] pressure sensors. Piezoresistive sensors convert pressure into changes in resistance and have a simple structure that allows for easy signal acquisition, which has garnered significant interest from researchers. Polydimethylsiloxane (PDMS) is widely used as a pressure-sensitive layer in flexible pressure sensors due to its flexibility, stability, and biocompatibility. However, PDMS materials fail to meet the high-performance requirements for sensing. To enhance sensitivity, many studies have introduced microstructures on the surfaces of PDMS [21,22,23], such as micro domes, micro pyramids, and biomimetic structures. These microstructures create significant local strain under low pressure, amplifying the pressure change signal. Despite their advantages, implementing these carefully designed microstructures can be complex and requires a complex preparation process. An alternative approach is to incorporate porous structures into PDMS materials [10,24,25,26], which can also improve sensor sensitivity. When an external force is applied, the pores in the PDMS foam are compressed until they close, significantly enhancing its deformation under pressure. This makes the pressure sensor, which is made of porous PDMS foam, more sensitive to pressure.

Currently, there are five main methods for preparing porous the PDMS materials: particle template foaming method, lotion foaming method, gas foaming method, solvent evaporation method, and printing method [27,28]. Among these, the particle template method is a relatively simple method for preparing porous PDMS foam. In this method, sacrificial particles, such as salt, sugar, or foam nickel, are uniformly dispersed in the PDMS prepolymer. Once the mixture solidifies, these template particles are removed through dissolution, resulting in a porous structure within the PDMS. The pore diameter and porosity of foam can be modified to suit different applications by changing the size and number of template particles. In contrast, the instability of the pore-forming mechanism of the lotion foaming method, gas foaming method, and solvent evaporation method results in higher requirements for the preparation process. Although 3D printing technology provides unprecedented design freedom for constructing PDMS materials with predetermined microstructures, its application is still limited by the rheological properties of the materials themselves. The viscosity of conventional PDMS prepolymers is too low, resulting in a liquid state requiring the addition of other materials to PDMS to increase its viscosity. This not only increases the complexity of the process, but may also involve post-processing operations, resulting in higher manufacturing costs.

PDMS is a non-conductive polymer, which makes it challenging to use directly as a pressure-sensitive material for a piezoresistive sensor. Consequently, enhancing the conductivity of PDMS foam is a critical issue. Numerous studies have added carbon-based conductive materials to PDMS, including carbon nanotubes [9,11,13,14], graphene [12,26], and carbon black [25,27]. Candle soot is also a common type of carbon black, primarily produced through the incomplete combustion of candles. It offers several advantages, including low cost and simple preparation. Liang et al. analyzed the substances present in the soot particles from both the internal flame and the tip of the flame, finding that the soot particles at the tip consist of 89 at.% carbon and 11 at.% oxygen [29]. This shows that candle soot contains conductive carbon, which can demonstrate excellent conductivity after being purified. To date, however, candle soot has not yet been used in PDMS pressure sensors.

In this paper, we have combined candle soot with porous PDMS foam to develop a flexible piezoresistive pressure sensor for the first time. Specifically, NaCl particles with different particle sizes were used as templates to regulate the pore structure of PDMS foam. Subsequently, the particles from the candle soot collected and purified from the flame tip were uniformly adhered to the surface of the porous PDMS foam using an ultrasonic-assisted immersion adsorption method, resulting in PDMS/candle soot (PDMS/CS) composite foam. Additionally, to enhance the stability and resistance to interference of the sensor, a PDMS film was used to completely encapsulate the PDMS/CS foam. This structure provides excellent airtightness, effectively isolating the sensitive elements from external environmental factors such as humidity and dust. Finally, the prepared sensor was used to detect finger movements such as pressing and bending, indicating its suitability for wearable electronic products.

## 2. Materials and Methods

### 2.1. Fabrication of Porous PDMS Foam

**Figure 1:**
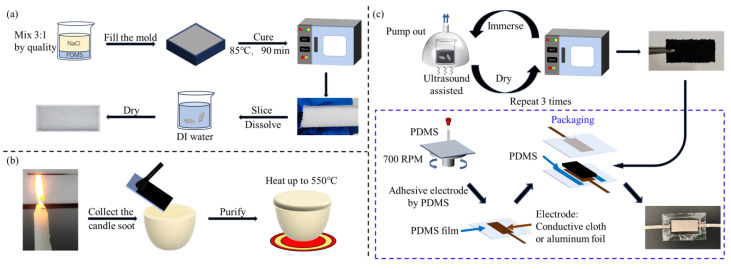
Sensor manufacturing flow chart. (**a**) Fabrication of porous PDMS foam, (**b**) preparation of candle soot, (**c**) fabrication of the pressure sensor.

The preparation process of porous PDMS foam is shown in Figure 1a. First, commercially available salt is screened through various mesh sizes to obtain uniform particle sizes. Screen NaCl with particle sizes of 100–200 μm, 200–300 μm, 300–400 μm, and 400–500 μm. Next, the PDMS (Sylgard184, Dow Corning, Midland, MI, USA) is prepared by mixing the PDMS prepolymer and curing agent in a ratio of 10:1 by weight. Add the screened salt as sacrificial particles to the PDMS mixed in the previous step, with a mass ratio of NaCl: PDMS of 3:1. Add 5 g of n-hexane to the PDMS and NaCl mixture to soften the PDMS. Stir the mixture for 10 min to ensure it is uniform. The combined PDMS and NaCl mixture is then poured into a mold with dimensions of 50 mm × 50 mm × 17 mm and placed in a vacuum chamber for 120 min to remove the n-hexane and air. Afterward, the mixture is cured in an oven at 85 °C for 90 min. Once cured, remove PDMS from the mold and cut it into 2 mm thick pieces. Then, immerse the block in deionized water for 12 h to dissolve the salt. Finally, porous PDMS foam is obtained after the drying process.

### 2.2. Preparation of Candle Soot

The preparation process of candle soot is shown in Figure 1b. First, hold the surface of the glass slide at the tip of the paraffin candle flame. The soot from the candle will gradually deposit a layer on the glass slide, causing it to turn black over time. After being exposed to the flame for 1 min, carefully remove the glass slide and allow it to cool naturally. Once it has cooled, use a brush to clean off the candle soot from the slide and transfer the residue into a ceramic container. Finally, heat the collected candle soot in the ceramic container to 550 °C to eliminate any impurities present in the soot.

### 2.3. Fabrication of the Pressure Sensor

PDMS/CS foam preparation: mix the purified candle soot with ethanol to obtain a candle ethanol solution with a concentration of 0.01 g/mL. Cut the PDMS foam material into pieces measuring 18 mm × 8 mm × 2 mm. Pump the air out of the PDMS foam to immerse it in the solution. After that, the beaker is placed into an ultrasonic cleaning machine for 10 min. Afterward, place the immersed composite material in an oven set at 80 °C and heat it for 20 min to dry the foam. To ensure complete adsorption of the candle soot, repeat the immersion and adsorption process three times.

Packaging process: PDMS was first spin-coated onto a glass substrate at 700 rpm for 45 s and subsequently cured at 90 °C for 30 min to form a thin film. Then, the electrode (conductive cloth and aluminum foil) coated with PDMS was tightly adhered to the PDMS film and heated to 90 °C for 20 min to ensure a tight bond. Next, the PDMS/CS foam was completely encapsulated by sandwiching it between two layers of the aforementioned PDMS films. To seal the interface between the two encapsulation layers, the junction was coated with uncured PDMS and cured at 90 °C for an additional 20 min, resulting in the final packaged sensor. Figure 1c illustrates the fabrication process of the porous PDMS.

### 2.4. Sensor Testing and Equipment

The pore structure of porous PDMS/CS foam was observed by field-emission scanning electron microscope (SU-8010, Hitachi, Japan). To test the pressure response performance of the sensor, a load was applied using a pressure sensor (hp–100, Handpi, China) and a pressure testing bench (HP500, Puyan, China). The real-time output signal of the sensor is collected through a digital multimeter (DMS855E, Rigol, China). In order to test the performance of the unpacked PDMS foam more conveniently, a glass sheet with a conductive cloth electrode is used as a temporary electrode.

## 3. Results and Discussion

### 3.1. Analysis of Porous PDMS/CS Foam

**Figure 2:**
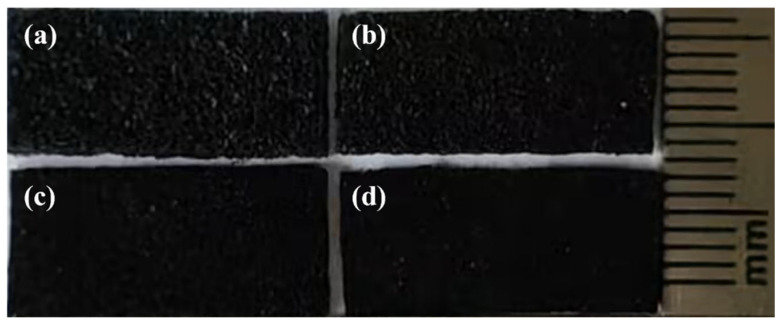
Cross sections of PDMS/CS foam with different apertures: (**a**) 400–500 μm, (**b**) 300–400 μm, (**c**) 200–300 μm, (**d**) 100–200 μm.

The porous PDMS/CS foam fabricated using NaCl particles of varying sizes is illustrated in Figure 2. Notably, during the preparation of the porous PDMS foam, it was observed that the NaCl particles and PDMS tend to stratify. And this stratification phenomenon is positively correlated with the size of the NaCl particles. This phenomenon can primarily be attributed to the differences in the sedimentation behavior of the particles within the mixed system. Larger NaCl particles tend to settle at the bottom of the PDMS mixture due to gravity, which causes the PDMS to rise and subsequently form a distinct layer on the surface. In contrast, smaller particles demonstrate better dispersibility and are less prone to settling, resulting in less pronounced surface layering. Additionally, using n-hexane as a diluent during the preparation process enhanced the fluidity of PDMS but also intensified the stratification phenomenon within the mixture. Under the same preparation conditions, the size of the NaCl particles significantly impacts the pore structure of the final foam. Specifically, larger particles accumulate in the bottom pores due to sedimentation, leading to higher overall porosity in the foam.

As shown in Figure 2, PDMS foam blocks are obtained by cutting the overall foam (similar to cutting bread). The cutting interface reveals an irregular structure, resulting in a highly rough surface on the foam. At the same time, the color of PDMS/CS foam exhibits a distinct visual difference with the pore size: the smaller the pore size, the darker the foam appears. This indicates that the surface roughness of foam changes with the pore size. This microstructure has a significant impact on the performance of the prepared sensors. The rough surface severely limits the effective contact area between the pressure-sensitive layer and the electrode under low pressure, resulting in a significant increase in the initial resistance of the sensor. As external pressure is applied, the microstructure of the foam surface deforms, increasing the contact area with the electrode and thereby reducing the contact resistance. This resistance change mechanism, regulated by the contact area, effectively expands the response to the pressure.

**Figure 3:**
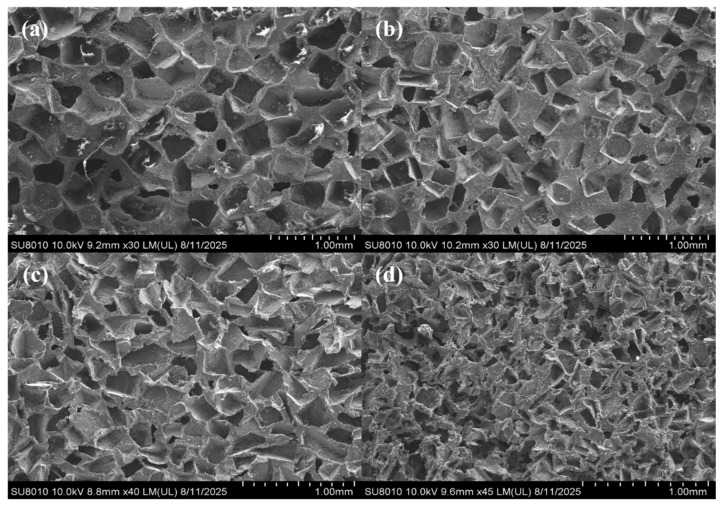
Cross-section SEM images of porous PDMS/CS foam prepared by adding different particles of NaCl: (**a**) 400–500 μm, (**b**) 300–400 μm, (**c**) 200–300 μm and (**d**) 100–200 μm.

In order to analyze the microstructure of PDMS/CS foam, its cross-section was examined using SEM, and the results are presented in Figure 3. The SEM images clearly show that the pore size of the foam is positively correlated with the size of the NaCl template particles used. The pore diameter of PDMS foam closely matches the size of NaCl particles. According to the SEM image, the porosity of 400–500 μm foam is about 72.779%, that of 300–400 μm foam is about 69.121%, that of 200–300 μm foam is about 67.342%, and that of 100–200 μm foam is about 63.511%. The data shows that the porosity of foam decreases with the decrease in its pore size. This result is consistent with previous results obtained based on the analysis of the PDMS stratification phenomenon. Furthermore, many irregular microstructures formed during the pore-forming process of NaCl particles can be observed on the surface of the foam. Notably, the PDMS/CS foam yielded clear SEM images without the need for gold sputtering. This indicates that a significant amount of candle soot was successfully and uniformly adsorbed on the surface of the foam, creating a continuous conductive network that greatly enhances the material’s conductivity.

**Figure 4:**
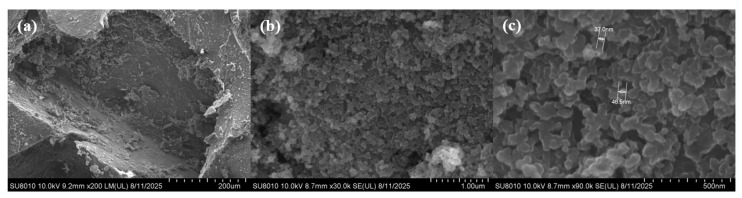
SEM images of PDMS/CS foam with a pore size of 400–500 μm after three adsorption operations: (**a**) low magnifi-cation, (**b**) medium magnification, and (**c**) high magnification.

To further investigate the adsorption status of candle soot in PDMS foam, the internal structure of the PDMS/CS foam with pore sizes ranging from 400 to 500 μm was carefully observed with SEM at a higher magnification. As shown in Figure 4a, candle soot forms a dense adsorption layer on the surface of PDMS/CS foam, which significantly enhances the conductivity of PDMS foam. It is worth noting that in the pores of the foam, a large number of relatively loose soot particles were also observed. This is mainly because PDMS/CS foam absorbed excessive candle soot during the preparation process, and the loose particles attached to the surface are easily dislodged by external forces and further accumulate in the pores. This phenomenon indicates that there is a saturation upper limit for PDMS foam to adsorb candle soot, and further increasing the adsorption capacity cannot effectively improve the sensor’s performance. Figure 4b shows the high magnification morphology of the dense soot layer on the surface of PDMS/CS foam, clearly revealing the tight physical connection between particles. The dense layer structure is stable and can effectively resist external interference, which is the key to building a stable conductive network. The above results collectively confirm that a large number of stable conductive pathways have been successfully constructed in the porous PDMS matrix through an ultrasound-assisted solution immersion method. In addition, as shown in Figure 4c, we also conducted a statistical analysis of the particle size of the prepared candle soot. The results showed that the particle size of particles collected in the tip area of the candle flame was relatively small, with a diameter mainly distributed between 30 and 50 nm.

### 3.2. Performance of PDMS/CS Foam Pressure Sensor

**Figure 5:**
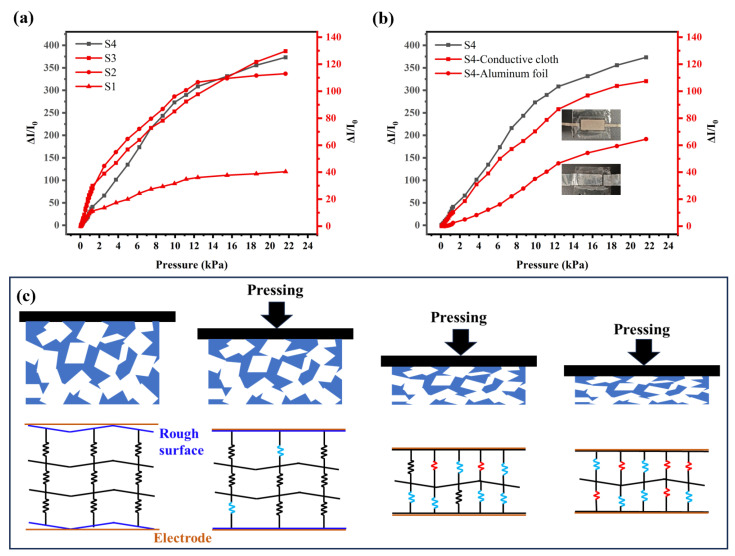
(**a**) Comparison diagram of the current change of unpacked (the electrode is conductive cloth) PDMS/CS foam with different apertures under pressure. (**b**) Sensor performance with S4 foam and different electrodes. (**c**) Schematic diagram of deformation and resistance change of PDMS/CS foam under different pressures.

The current change curve of the PDMS/CS foam pressure sensor measured under pressure is shown in Figure 5. Porous PDMS composite materials prepared using NaCl particles of sizes 100–200 μm, 200–300 μm, 300–400 μm, and 400–500 μm were named S1, S2, S3, and S4, respectively. Because candle soot is a zero-dimensional nanomaterial with a diameter of approximately 30–50 nm, its ability to construct a continuous conductive network is relatively weak. At the same time, the rough surface structure of porous PDMS foam leads to the extremely high initial resistance of PDMS/CS foam without external pressure, which makes it difficult to measure accurately. It is necessary to adsorb a sufficient amount of candle soot, as far as possible, to enhance the conductivity of the device. However, there is a clear upper saturation limit for the adsorption amount of candle soot on the surface of PDMS foam. The selected PDMS/CS foam is soaked and adsorbed with candle soot more than three times to facilitate the measurement of resistance and current changes of the foam. There is no significant difference in the performance of foam soaked and absorbed in candle soot three or more times. In addition, the PDMS/CS foam in Figure 5a uses the temporary electrode of conductive cloth.

The sensitivity of the above sensors is shown in Table 1 and Table 2. The sensitivity of a pressure sensor is defined as (ΔI/I_0_)/ΔP, where ΔI is the current change of the sensor, I_0_ is the reference current of the sensor under a specified external pressure, and ΔP is the pressure applied to the sensor.

As a pressure-sensitive material, the deformation process of PDMS/CS foam under pressure can be divided into three stages, and the corresponding pressure response mechanism is shown in Figure 5c. In the first stage, the applied small pressure mainly causes the deformation of the microstructure of the foam surface, thus rapidly increasing the effective contact area between the foam and the electrode. This causes a drastic change in the foam resistance, giving the sensor a very high sensitivity at this stage. In the second stage, as the pressure continues to increase, the pores in the foam gradually close, forming more point-to-point conductive paths. Thanks to the excellent compressibility of the porous structure, the sensor can still maintain high sensitivity during this process. However, when the deformation enters the third stage, the pores in the foam have basically closed, and the resistance change mainly depends on the further compression of the PDMS material itself. Obviously, the sensitivity of the sensor significantly decreases at this stage, and its ability to distinguish pressure changes also weakens accordingly. The effective detection range of the sensor prepared in this study mainly focuses on the first two stages of foam deformation. This is because, as the pressure continues to increase and approaches the third stage, the sensor exhibits abnormal piezoresistive response characteristics. The resistance value no longer follows the traditional monotonic decreasing law, but exhibits unstable fluctuations, and even increases with increasing pressure rather than monotonically. This anomalous phenomenon can be attributed to the specific structure of the sensor. Because the conductive medium, candle soot, mainly adheres to the surface of the PDMS skeleton through physical adsorption, the adsorption layer structure is easily damaged under severe mechanical deformation caused by high pressure. Specifically, the excessive extrusion of the foam skeleton destroyed the conductive network on the surface, leading to the fracture of the conductive path. The destruction of this microstructure directly leads to unstable changes in resistance and non-monotonic rise phenomena, which make the sensor unsuitable for high-voltage applications.

As shown in Table 1, the S4 foam with the largest pore size exhibits the best performance, with a high sensitivity of 32.90 kPa^−1^ in the range of 46 Pa to 1.28 kPa. Additionally, it maintains a strong sensitivity of 25.52 kPa^−1^ within the range of 1.28–12.39 kPa. However, the porous structure becomes significantly closed in the range of 12.39–21.65 kPa, leading to a sharp decrease in sensitivity to 7.08 kPa^−1^. Table 1 and Figure 5a show that the sensitivity of foam decreases with the decrease in pore size. This occurs because larger pore sizes create a rougher and more uneven foam surface, reduce the initial contact area with the electrode, and consequently lower the initial current. Additionally, PDMS foam made with larger salt particle sizes has a higher proportion of pores, making it more sensitive to pressure. These factors contribute to the optimal performance of the PDMS/CS foam with pore sizes ranging from 400 to 500 µm. As the pores of PDMS/CS foam decrease, its closing pressure gradually diminishes, and the response range with higher sensitivity becomes narrower.

In the experiment, it was observed that prolonged exposure of PDMS/CS foam to air resulted in increased resistance, which consequently reduced sensitivity. Therefore, a thin film prepared by PDMS is used as the external packaging of the sensor to isolate the external environment. Figure 5b shows the performance of the packaged S4 sensor, with a significant decrease in sensitivity after packaging. The sensitivity of the packaged sensor is presented in Table 2. This decline in sensitivity is primarily due to the structure of the conductive cloth. The structure of the conductive cloth reduces the contact area between the electrode and the PDMS/CS foam under low pressure. In contrast, under high pressure, the internal gap of the cloth is filled with PDMS/CS foam, resulting in a significant increase in the contact area. This increased contact area magnified the resistance change of PDMS/CS foam. Therefore, the PDMS adhesive used in the packaging process fills the gaps in the conductive fabric, further reducing the maximum contact area of the electrodes. Additionally, we also attempted to use aluminum foil as the electrode for the sensor. However, the aluminum foil electrode is not soft enough to fully contact the PDMS/CS foam, resulting in poor performance of the sensor. The final sensor uses relatively good conductive cloth as the electrode. It demonstrates a sensitivity of 8.92 kPa^−1^ within the range of 0.077–1.28 kPa and a sensitivity of 6.78 kPa^−1^ in the range of 1.28–12.39 kPa. Similarly, the sensitivity significantly decreases to 2.24 kPa^−1^ when the pressure is in the range of 12.39–21.65 kPa. Although the performance of the sensor decreases after packaging, the fully enclosed design enhances its resistance to external interference, making it easier to apply. As shown in Table 2, compared with other resistance PDMS pressure sensors, the sensor we prepared has excellent sensitivity under low pressure.

### 3.3. Application Tests

**Figure 6:**
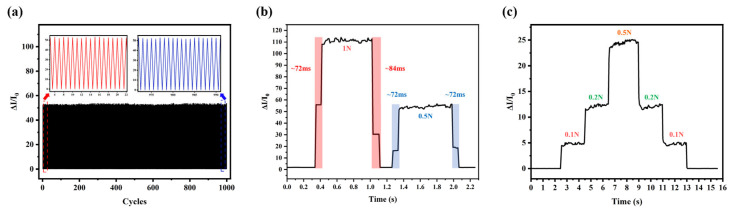
(**a**) The sensor undergoes 1000 pressure load tests under a pressure of 1 N. (**b**) Response of current change of sensors under 0.5 N and 1 N pressures. (**c**) The current variation of the prepared sensor under stepped pressure.

We selected a sensor with conductive cloth as the electrode and encapsulated it in PDMS film for application testing. The sensor’s performance was evaluated over 1000 loading/unloading cycles under a pressure of 1 N, as shown in Figure 6a. The results demonstrate that the sensor maintains stability during repeated applications of pressure. Additionally, the response curve of the sensor was also measured, as shown in Figure 6b. Due to the sampling rate of the measuring equipment, the response signal in the figure shows a clear step-like pattern. The response time and recovery time of the sensor under different pressures are both about 72 ms, which is comparable to the performance of most sensors currently reported. Figure 6c shows that the current variation of the sensor under continuous stepped pressure is significant and relatively stable. This result indicates that the sensor possesses excellent pressure resolution and reliability, making it suitable for actual pressure detection applications.

**Figure 7:**
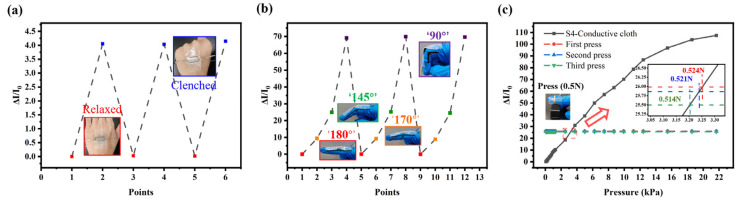
(**a**) The change in sensor current when the palm is clenched and relaxed. (**b**) The change in sensor current when the finger is bent. (**c**) The sensor detects current changes when pressing a keyboard key.

In Figure 7a, the sensor is securely attached to the back of the hand using tape. Notably, significant changes in electrical current were observed when the palm was clenched and relaxed. Furthermore, as shown in Figure 7b, the sensor can detect changes in finger bending. It effectively captures the bending motion of fingers in practical applications and is highly sensitive to current changes when detecting slight finger bending. We also assessed the accuracy of the pressure measurement sensor described in this article. The sensor was attached to a finger while pressing the keyboard to measure the current generated during the press. This was compared to the current pressure curve of the S4-conductive cloth sensor. The average measured pressure during keyboard pressing was found to be 0.519 N, which is very close to the trigger pressure of about 0.5 N (3.13 kPa). These practical application test results confirm the accuracy and reliability of the sensor in measuring small pressure changes.

## 4. Conclusions

In summary, a novel, flexible piezoresistive sensor based on porous PDMS/candle soot foam has been demonstrated. The fabrication process is quite simple and cost-effective. Due to its porous structure, rough surface, and the distinctive characteristics of candle soot, the proposed sensor shows exceptional sensitivity within the low-pressure range. Specifically, sensors with apertures between 400 and 500 μm show a sensitivity of 8.92 kPa^−1^ under the low-pressure conditions of 0.1–1.28 kPa and maintain a high sensitivity of 6.78 kPa^−1^ within the range of 1.28–12.39 kPa. Compared with sensors prepared using other common conductive materials, this method shows comparable or even more advantages in low-voltage detection performance. Meanwhile, application tests indicate that this sensor has great potential for practical use in various human motion detection situations. In the future, we will further improve the packaging of sensors to reduce their impact on performance.

## Figures and Tables

**Table 1:** Sensitivity of porous PDMS foam with different pore sizes.

	S4	S3	S2	S1
Sensitivity (kPa^−1^) 0.046–1.28 kPa	32.90	25.93	23.36	9.57
Sensitivity (kPa^−1^) 1.28–12.39 kPa	25.52	6.12	6.79	2.42
Sensitivity (kPa^−1^) 12.39–21.65 kPa	7.08	3.47	0.71	0.65

**Table 2:** Performance comparison with other flexible pressure sensors.

Sensor	Range (kPa)	Sensitivity (kPa^−1^)	Response/Recovery Time (ms)
S4-Conductive cloth (this work)	0.077–1.28	8.92	72/72
	1.28–12.39	6.78	
	12.39–21.65	2.24	
S4-Aluminum Foil (this work)	0.41–1.28	2.5	——
	1.28–11.15	4.08	
	11.15–21.65	1.91	
PDMS/CB [25]	0–100	0.52	46/46
PDMS/ AgNWs [30]	0–0.05	2.588	10/20
	0.05–5	0.131	
	5–20	0.015	
PDMS/AG [31]	0–15	5.9	42/53
rGO@MWCNTs/ PDMS [32]	0–28.8	1.62	61/40
	28.8–200	0.18	
PDMS/FIBER@ MWCNT [33]	0–20	4.07	41/60
	20–70	0.69	
PDMS/CB & CNT [34]	0–25	7.1	175/165
	25–135	2.96	
	135–350	1.09	
PDMS/CPs & CNPs [22]	0.02–600	26.6	40/20

## Data Availability

Data will be made available on request.
